# Transarterial Embolization Alone Versus Drug-Eluting Microspheres Chemoembolization for Hepatocellular Carcinoma (RAD-18-TAcE): A Multicenter Randomized Clinical Trial

**DOI:** 10.1007/s00270-026-04487-3

**Published:** 2026-06-09

**Authors:** Cristina Mosconi, Makoto Taninokuchi Tomassoni, Sara Zanella, Alexandro Paccapelo, Giovanni Marasco, Simone Gusmini, Francesca Ratti, Diego Palumbo, Sandro Sironi, Riccardo Muglia, Marco Mario Edgardo Fronda, Marco Calandri, Paolo Fonio, Paolo Marra, Andrea Doriguzzi Breatta, Alessandro Cucchetti, Francesco De Cobelli, Matteo Renzulli

**Affiliations:** 1 Department of Radiology, IRCCS Azienda Ospedaliero-Universitaria Di Bologna, Via Albertoni 15, Bologna, Italy; 2 Epidemiology and Statistics, IRCCS Azienda Ospedaliero-Universitaria Di Bologna, Bologna, Italy; 3 Department of Medical and Surgical Sciences-DIMEC, Alma Mater Studiorum–University of Bologna, Bologna, Italy; 4 Department of Radiology, IRCCS San Raffaele Hospital, Milan, Italy; 5 Hepatobiliary Surgery Division, IRCCS San Raffaele Hospital, Milano Vita Salute San Raffaele University, Milan, Italy; 6 Department of Radiology, IRCCS San Raffaele Hospital, Milano Vita Salute San Raffaele University, Milan, Italy; 7 School of Medicine and Surgery, University of Milano-Bicocca, Piazza Dell’Atteneo Nuovo 1, Milan, Italy; 8 Department of Radiology, ASST Papa Giovanni XXIII Hospital, Piazza OMS 1, Bergamo, Italy; 9 Department of Diagnostic Imaging and Interventional Radiology, AOU Città Della Salute E Della Scienza Di Torino, Corso Bramante 88, 10128 Turin, Italy; 10 Department of Surgery, Morgagni–Pierantoni Hospital, Forlì, Italy

**Keywords:** Hepatocellular carcinoma, Transarterial embolization, Drug-eluting microspheres embolization, Chemoembolization, Randomized clinical trial

## Abstract

**Purpose:**

To compare transarterial embolization (TAE) and drug-eluting microspheres transarterial chemoembolization (DEM-TACE) in the treatment of hepatocellular carcinoma (HCC) through a non-inferiority randomized clinical trial.

**Materials and Methods:**

Patients diagnosed with unresectable HCC were randomly assigned to either the TAE or DEM-TACE group. The primary endpoint was time to progression (TTP), and secondary endpoints included overall survival, hospitalization stay, tumor response rates, and severe adverse events (SAE).

**Results:**

At the end of the trial, a total of 111 patients (56 TAE and 55 DEM-TACE) were enrolled between 2019 and 2022. The median TTP was similar between TAE and DEM-TACE (7.56 vs. 7.98 months, *p* = 0.432). In patients with confirmed disease progression, the mean TTP was 6.68 vs. 8.43 months for TAE and DEM-TACE, respectively, with a mean difference of 1.75 months (95% CI − 1.47–4.97), falling within the predefined equivalence range; two one side test (TOST) analysis confirmed equivalence (*p* < 0.001 and *p* = 0.022 for lower and upper bounds. Overall survival, tumor response rates, and adverse events were also not statistically different between groups (*p* > 0.05 for each outcome). Finally, Days of hospitalization were not statistically different (median 4 days for both arms, *p* = 0.638).

**Conclusion:**

TAE and DEM-TACE had comparable results in the treatment of early and intermediate stage unresectable HCC.

**Graphical abstract:**

**Figure:**
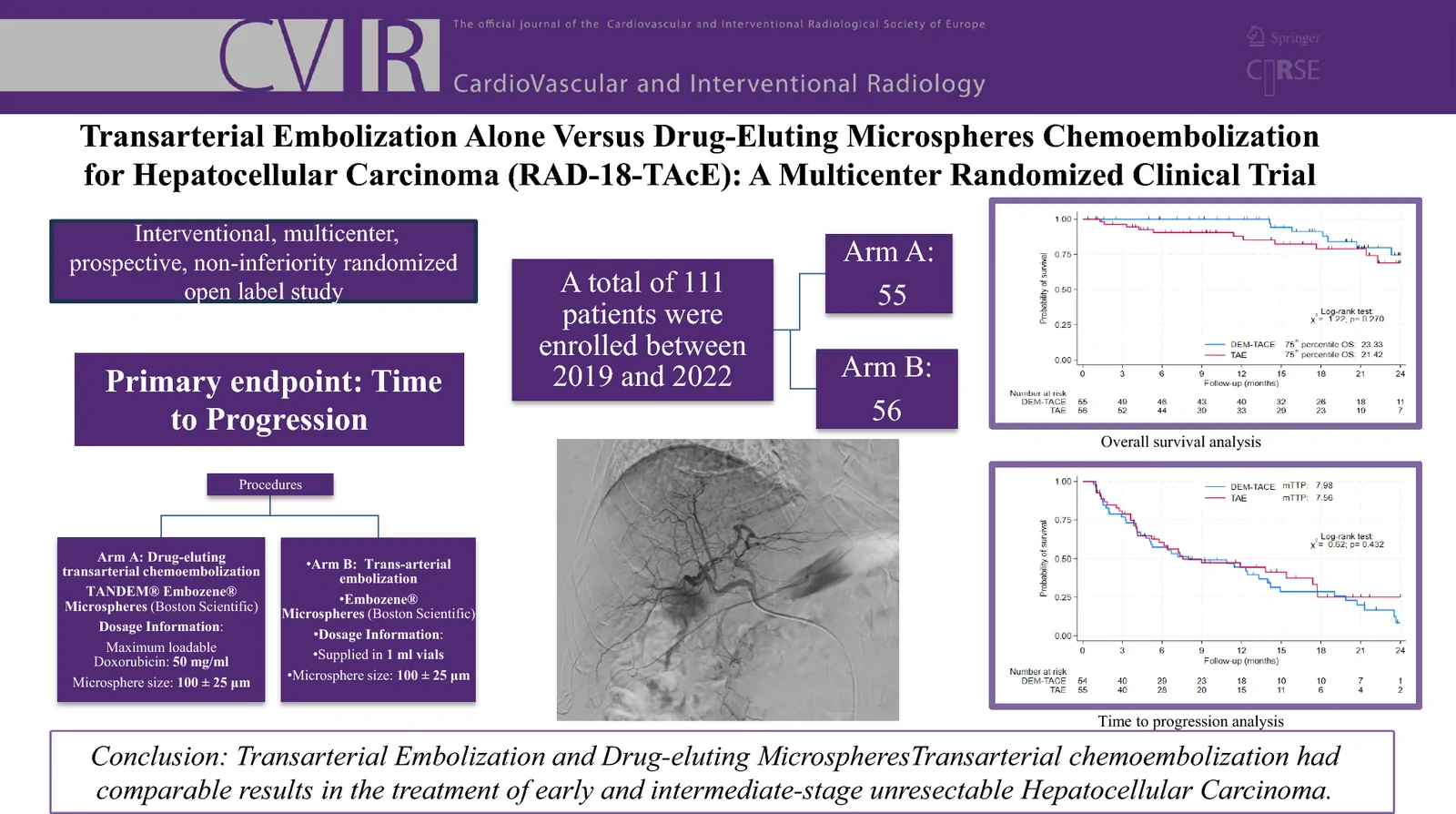

**Supplementary Information:**

The online version contains supplementary material available at https://doi.org/10.1007/s00270-026-04487-3.

## Introduction

Hepatocellular carcinoma (HCC) presents a considerable challenge to healthcare systems worldwide, with its increasing incidence and high mortality rates [1]. Among the various therapeutic modalities available for this tumour, transarterial interventions represent a cornerstone approach, offering minimally invasive localized treatment with good outcomes [2–4].

Transarterial chemoembolization (TACE) is the most adopted endovascular treatment for unresectable HCC, using the delivery of chemotherapeutic agents directly to the tumor while simultaneously inducing ischemia through embolization [5]. Drug-eluting microspheres transarterial chemoembolization (DEM-TACE) offers sustained drug release and more controlled embolization, resulting less toxic [6] but without differences in patient survival than conventional chemoembolization (c-TACE)[7]. Since its introduction, DEM-TACE has become the standard in many centers. Transarterial embolization (TAE) is an embolic technique without the concurrent use of chemotherapeutic agents, demonstrating good efficacy in achieving tumor devascularization [8]. Despite the growing utilization of DEM-TACE, questions persist regarding its comparative efficacy and safety relative to conventional TAE. While DEM-TACE offers theoretical advantages by its double chemotherapeutic and embolic action, whether these translate into meaningful clinical outcomes remains uncertain. In fact, there is no clear evidence of benefits in associating anticancer agents with simple embolization to date. Additionally, it is still unclear whether side effects following embolization procedures are related to the embolization itself, the addition of the drug, or both components. Finally, DEM-TACE is more expensive than TAE. Thus, given the current attention on cancer-related health care cost control, the identification of opportunities for cost savings in HCC treatments of an increasingly common cancer can improve healthcare.

The present study aims to compare first-line DEM-TACE and TAE on unresectable HCC under the hypothesis that the addition of drug to embolization with small size microspheres is not associated with a difference regarding time to progression (TTP).

## Materials and Methods

### Study Design

This interventional, multicenter, prospective, non-inferiority randomized open label study aimed to compare the efficacy and safety of TAE and DEM-TACE in patients with unresectable HCC. The trial was conducted in accordance with ethical standards and received approval from the institutional review boards. The study was registered at www.clinicaltrials.gov (NCT04803019).

### Selection Criteria

Patients were selected according to the inclusion and exclusion criteria summarized in Table 1.

**Table 1:** Inclusion and exclusion criteria of the study

*Inclusion Criteria*	*Exclusion Criteria*
HCC diagnosis according to AASLD criteria released prior to the study start. Age ≥ 18 years. Patients able to understand the participation to the study and to sign a written consent. HCC unsuitable for potentially curative treatment and/or recurred after resection/ablation diagnosed according to the AASLD criteria. No previous treatment in target lesions (prior treatments including resection on non-target lesions were accepted). Child–Pugh class A or B (max score 7). ECOG Performance Status (PS) < 2. Target lesion measurable according to mRECIST. m-HAP-II classes B or C fulfilment. UNOS/TMN stage T1-4a.	Infiltrative HCC; neoplastic branch or main portal vein invasion. Equivocal hepatic lesion/s; Advanced liver disease (bilirubin levels > 2.5 mg dl-1, albumin < 30 g l-1, platelets < 50 × 109/L, INR > 1.5); ascites and/or F3 esophageal varices. Other tumors in the previous 5 years. Technical contraindications to arteriography or TACE/TAE.

The m-HAP-II classes B or C fulfilment and the UNOS/TNM stage were applied for stratification of the randomization to obtain similar prevalences of m-HAP-II classes B/C and of UNOS/TNM stages from T1 to T4a in the two arms.

### Endpoints

The primary endpoint was time-to-progression (TTP), defined as the time interval between the initiation of treatment and the objective progression of disease, as determined by the modified Response Evaluation Criteria in Solid Tumors (mRECIST) criteria[9]. Secondary endpoints included a safety analysis through the evaluation of serious adverse events (SAEs) (from grade 3 and above according to CIRSE standards of Practice classification [10]), comparison of tumor radiological response and overall survival (OS), and, finally, the hospitalization days of DEM-TACE versus TAE.

### Sample Size and Randomization

The study was designed, with respect to the primary endpoint, as an equivalence trial between DEM-TACE and TAE. The reference value for TTP in the DEM-TACE arm was set at 9 months, based on the results of our previous multicenter experience [11]. It was expected that the standard deviation of TTP would have not exceeded 6 months, assuming careful patient selection consequent to enrollment criteria and stratification randomization. The equivalence margin was set at no more than 5 months between the two arms. Accordingly, using appropriate statistical formulas, each arm would have included 69 patients (alpha: 0.05; 1-beta: 0.80). Considering a 10% dropout rate, the final sample size for each arm would have been 77 patients, for a total of 154 participants.

### Treatments

Both treatment arms received arterial embolization of the branches that vascularize HCC/s but only patients in the DEM-TACE arm (Arm A) received intra-arterial chemotherapy (doxorubicin). Both treatments were repeated “on demand” after demonstration to the imaging of the presence of residual vital tumor or in case of intrahepatic distal recurrence at follow-up. The level of catheterization during embolization (lobar, segmental, or subsegmental) was selected based on case complexity, with a super-selective approach prioritized whenever technically feasible.

Arm A (DEM-TACE chemoembolization with microspheres): The chemotherapy used in this arm was doxorubicin carried into the tumor by Embozene TANDEM® (Boston Scientific) microspheres. TANDEM® Embozene microspheres are made of non-resorbable, biocompatible, hydrogel microspheres, subjected to precision calibration and coated with an inorganic perfluorate polymer (Polyzene®-F). The microspheres were loaded with doxorubicin according to manufacturer indication. The maximum loadable amount on the hydrospheres was 50 mg of Doxorubicina for ml. The maximum injectable dose of Doxorubicin for each treatment was 150 mg and therefore the maximum amount of microspheres used for each treatment was 3 ml. The size of microspheres used in this study was up to 100 ± 25 μm.

Arm B (TAE embolization with microspheres). The TAE was performed with Embozene microspheres (Boston Scientific). The same size (100 μm) was used for both groups.

### Imaging Assessment and Follow up

Tumor response and TTP were assessed according to mRECIST. Contrast-enhanced multiphasic computed tomography (CT) or magnetic resonance imaging (MRI) was used for imaging evaluation, depending on local availability, in accordance with European Association for the Study of the Liver (EASL) guidelines[3]. Imaging follow-up was scheduled at 1, 3, 6, 9, 12, 15, 18, 21, and 24 months after treatment initiation. Image assessments were performed at each participating site by experienced radiologists according to institutional standards.

### Statistical Analysis

Patient demographic and clinical characteristics were reported as frequencies and percentages for categorical variables and as mean ± standard deviation or median and interquartile range for continuous variables. In order to investigate the presence of difference in baseline population characteristics between the two groups, we performed the Chi-squared or Fisher test for categorical data. For continuous variables, after assessing the distributional normality of continuous variables with the Shapiro–Wilk test, we performed a t-test on the log-normalized variable, or Mann–Whitney U test if no log-normalization was possible. TTP and OS data were analysed using the Kaplan–Meier procedure. Furthermore, an equivalence test using the Two One-Sided Tests (TOST) approach was conducted to assess whether the difference in mean TTP between the two groups was small enough to consider the treatments equivalent. As stated before, the equivalence range was set from − 5 to + 5 months. The hazard ratios (HRs) were computed together with their 95% confidence intervals (95%CIs). Two tailed *p* values < 0.05 were considered statistically significant. The analyses were carried out using IBM SPSS 27.0 (Armonk, NY: IBM Corp).

## Results

### Patient Selection and Characteristics

A total of 111 patients were enrolled in the trial between December 2019 and December 2022, with 56 patients in the TAE group and 55 in the DEM-TACE group. Our previous sample size calculation established 69 patients per group, nevertheless only 56 in the TAE arm and 55 in the DEM-TACE arm were included due to lack of recruitment (mainly due to COVID-19 pandemic).

Four centers were included in this study, centers involved and patients’ characteristics are summarized in Table 2 and 3. As can be observed, the two groups were comparable in regard to baseline characteristics. In the DEM-TACE group, the mean age was 69.7 years. The median maximum tumor dimension was 20.0 mm with a median of number of nodules of 1. In this group there were 31 patients with a MELD score between 6 and 9, 21 between 10 and 13, 3 over 14. In the TAE group, the mean age was 67.4 years, the median maximum tumor dimension in this group was 23.0 mm with a median of number of nodules of 1.5.. In this group there were 21 patients with a MELD score between 6 and 9, 28 between 10 and 13, 7 over 14. Re-treatment rate and level of catheterization during embolization on each group is described in Table 4, With no statistical differences between both groups.

**Table 2:** Patients characteristics and the division between each arm

	DEM-TACE		TAE		*P-value*
*n*	55	49.5%	56	50.5%	
Gender^1^					0.580
Male	49	89.1%	47	83.9%	
Female	6	10.9%	9	16.1%	
Site^1^					0.856
IRCCS AOUBo–Bologna	37	67.3%	40	71.4%	
AOU S. Giovanni Battista–Torino	8	14.5%	8	14.3%	
Ospedale Papa Giovanni XXIII–Bergamo	4	7.3%	2	3.6%	
Istituto Scientifico San Raffaele–Milano	6	10.9%	6	10.7%	
Child-pugh^1^					0.284
5	26	47.3%	27	48.2%	
6	19	34.5%	13	23.2%	
7	10	18.2%	16	28.6%	
MELD^1^					0.105
6–9	31	56.4%	21	37.5%	
10–13	21	38.2%	28	50.0%	
14 +	3	5.5%	7	12.5%	
ECOG^1^					0.495
0	55	100.0%	54	96.4%	
1	0	0.0%	2	3.6%	
BCLC^1^					0.858
0	13	23.6%	11	19.6%	
A	29	52.7%	32	57.1%	
B	13	23.6%	13	23.2%	
INR ≥ 1.7^1^	0	0.00%	2	3.60%	0.495
Bilirubin^1^					0.591
− < 2	47	87.00%	46	82.10%	
−2–3	6	11.10%	7	12.50%	
− > 3	1	1.90%	3	5.40%	
AFP ≥ 400^1^	2	5.00%	4	11.10%	0.414
Age^2^	71.0	61.2–77.0	67.8	59.3–75.2	0.221
Weight^2^	80.0	73.0–89.0	85.5	75.3–100.5	0.101
Height^2^	170.0	165.0–176.0	173.0	163.5–180.0	0.435
Albumin^2^	3.8	3.5–4.0	3.7	3.4–3.9	0.24
Creatinine^2^	0.8	0.7–1.0	0.8	0.7–1.0	0.681^A^
Platelet^2^	104.0	58.0–154.0	84.5	50.8–129.3	0.191^A^
AST^2^	31.0	23.5–48.0	42.0	30.0–62.0	0.068^A^
ALT^2^	26.0	17.5–43.5	27.0	20.0–48.0	0.254^A^
PCR^2^	0.3	0.1–0.7	0.5	0.3–0.8	0.054^A^
Neutrophil^2^	64.0	58.2–69.7	62.9	52.8–70.9	0.366
Lymphocyte^2^	22.1	18.9–30.6	24.6	17.9–32.7	0.378
Days of hospitalization^2^	4.0	3.0–6.0	4.0	3.0–6.0	0.638^B^
Max dimension (mm)^2^	20.0	16.0–32.0	23.0	16.0–32.0	0.829^B^
Number of Nodules (n)^2^	1.0	1.0–2.0	1.5	1.0–3.0	0.529^B^
UNOS-TNM^1^					0.910
T1	17	30.90%	14	25.00%	
T2	29	52.70%	33	58.90%	
T3	9	16.40%	9	16.10%	
UNOS staging^1^					0.913
I	17	30.90%	14	25.00%	
II	28	50.90%	31	55.40%	
III	10	18.20%	11	19.60%	
Total	55	100.00%	56	100.00%	
m-HAP II^1^					0.130
B	30	54.50%	22	39.30%	
C	25	45.50%	34	60.70%	

^1^Frequencies and percentages, ^2^Median and Interquartile Range, ^*A*^*t-test performed on the normalized variable*, ^*B*^*Mann–Whitney U test*

**Table 3:** Patients etiology distribution

	DEM-TACE		TAE		Total		*P-*value
Etiology							
HBV +							0.333
Absent	43	78.2%	48	85.7%	91	82.0%	
Present	12	21.8%	8	14.3%	20	18.0%	
HCV +							0.444
Absent	30	54.5%	35	62.5%	65	58.6%	
Present	25	45.5%	21	37.5%	46	41.4%	
HIV +							1.000
Absent	55	100.0%	55	98.2%	110	99.1%	
Present	0	0.0%	1	1.8%	1	0.9%	
Alcool							0.228
Absent	40	72.7%	34	60.7%	74	66.7%	
Present	15	27.3%	22	39.3%	37	33.3%	
NAFALD							0.094
Absent	48	87.3%	54	96.4%	102	91.9%	
Present	7	12.7%	2	3.6%	9	8.1%	
Cryptogenetics							0.094
Absent	45	81.8%	52	92.9%	97	87.4%	
Present	10	18.2%	4	7.1%	14	12.6%	
Other							0.143
Absent	43	78.2%	36	64.3%	79	71.2%	
Present	12	21.8%	20	35.7%	32	28.8%

**Table 4:** Treatment type and retreatment rate

	DEM-TACE		TAE		Total		*P*-value
Retreatments							0.324
No	33	60.0%	39	69.6%	72	64.9%	
Yes	22	40.0%	17	30.4%	39	35.1%	
Retreatments within 6 months							0.364
No	41	74.5%	46	82.1%	87	78.4%	
Yes	14	25.5%	10	17.9%	24	21.6%	
Subsegmental treatment	43	78.2%	46	82.1%	89	80.2%	0.641
Segmental treatment	12	21.8%	10	17.9%	22	19.8%	

### Time to Progression

**Fig. 1:**
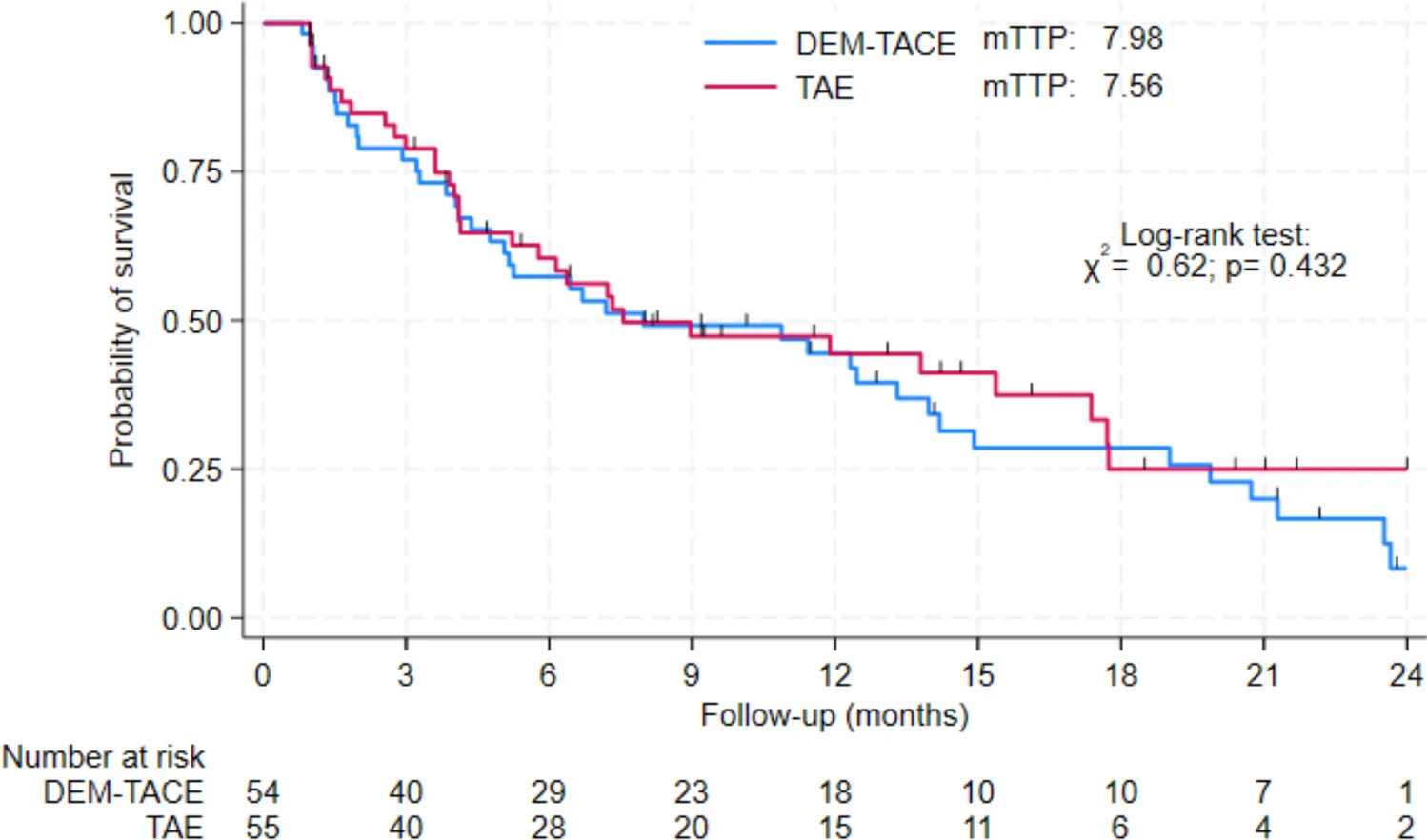
Time to progression analysis

The median follow-up was 18.5 months. Two patients (one per arms) were excluded from TTP calculation because they didn’t undergo any radiological follow-up, one being transplanted and one dying before the first follow-up. In terms of TTP, the two treatments provided similar results, with a median of 7.98 in the DEM-TACE group and 7.56 months in the TAE, (*p* = 0.432) (Fig. 1).

Considering only patients with confirmed disease progression (*n* = 74, being 41 and 33 in DEM-TACE arm and in TAE arm respectively), a mean TTP of 8.43 months was calculated in the group subjected to DEM-TACE and 6.68 months in the group subjected to TAE, resulting in a mean difference of 1.75, with a 95% confidence interval for the difference ranging from − 1.47 to + 4.97. The two-tailed *p*-value was 0.281, meaning there was no evidence to reject the null hypothesis that the two treatments were equivalent. Furthermore, the confidence interval for the mean difference falls within the equivalence range, supporting the conclusion that the treatments were equivalent. The results of the TOST showed *p* < 0.001 for lower TOST and *p* = 0.022 for upper TOST.

### Post-hoc Power Analysis

With the mean TTP being 8.43 ± 7.43 months in the 41 patients with confirmed disease progression in the DEM-TACE arm and 6.68 ± 6.18 months in the 33 patients with confirmed disease progression in TAE arm, using two one-sided, two-sample, unequal-variance (Welch’s) t-test, with an overall Type I error rate (*α*) of 0.05, the power to detect the observed mean difference of 1.75 was 0.65.

### Adverse Events

In terms of safety, the SAEs encountered were very small in number and were summarized in Table 5. In particular, two SAEs were found in the DEM-TACE group possibly correlated to the procedure (pancreatitis and hepatic abscess), whereas only one SAE was found in the TAE group (Gastric hemorrhage).

**Table 5:** Serious adverse effects distribution

	Treatment		
	DEM-TACE	TAE	Total
Gastrointestinal disorders:			
Pancreatitis	1 (1,8%)	0	1(0.9%)
Gastric hemorrhage	0	1	1(0.9%)
Infective complications:			
Hepatic abscess	1 (1,8%)	0	1(0.9%)

### Overall Survival and Tumor Response Assessment

**Fig. 2:**
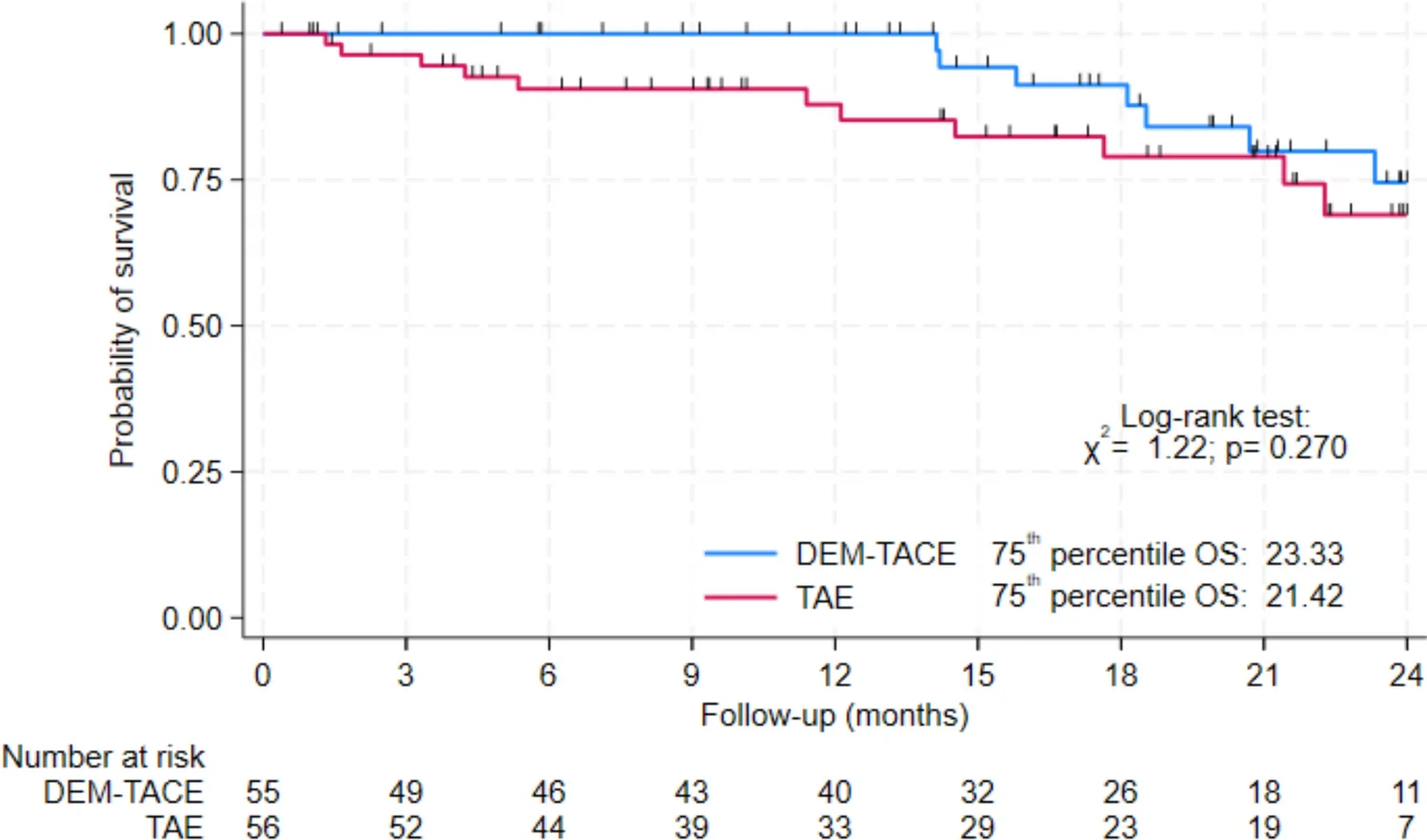
Overall survival analysis

OS and the radiological response of the tumor assessed using dedicated criteria (mRECIST) was evaluated in terms of Best Overall Response at 6 months and compared in the two treatments. As reported in the following tables, in the DEM-TACE group, 57.4% of patients achieved a complete response, 27.8% a partial response, 1.9% stable disease, and 13.0% progressive disease. In the TAE group, 49.1% achieved a complete response, 38.2% a partial response, 3.6% stable disease, and 9.1% progressive disease. The objective response rate was 85.2% for DEM-TACE and 87.3% for TAE (p = 0.788), while disease control was 87.0% and 90.9%, respectively (*p* = 0.556). The OS analysis showed a mean survival of 24.40 months for DEM-TACE and 22.00 months for TAE, with no statistically significant difference between the groups (*p* = 0.270). These results suggest similar efficacy and survival outcomes between DEM-TACE and TAE treatments (Table 6and Fig. 2).

**Table 6:** Summary of best overall response in both groups

Best Overall Response after 6 months	DEM-TACE		TAE		Total		P-value
Complete response	31	(57.4%)	27	(49.1%)	58	(53.2%)	0.445^A^
Partial response	15	(27.8%)	21	(38.2%)	36	(33.0%)	
Stable disease	1	(1.9%)	2	(3.6%)	3	(2.8%)	0.586^B^
Progressive disease	7	(13.0%)	5	(9.1%)	12	(11.0%)	
Total	54	(100.0%)	55	(100.0%)	109	(100.0%)	
Objective response rate	46	(85.2%)	48	(87.3%)	94	(86.2%)	0.788
Disease control	47	(87.0%)	50	(90.9%)	97	(89.0%)	0.556

^A^Complete Response versus others, ^B^Overall distribution

### Hospitalization Days

The average days in the TAE group was 5.20 and 4,62 in the DEM-TACE group. This factor was evaluated considering that, given the same effectiveness of the two treatments highlighted by the previous points, there was no statistically significant difference in terms of hospitalization days between DEM-TACE and TAE (both medians were 4 days, *p* = 0.638).

## Discussion

Conventional TACE was considered the standard treatment for intermediate-stage BCLC [4], nevertheless, this procedure has a considerable downfall, which is the lack of technique standardization, leading to an inexact outcome comparison between patients and different centers. In the last years, DEM-TACE has been introduced as a variant that gives comparable OS rates and TTP, with more tumour-selective drug delivery and permanent embolization, facilitating procedure standardization[6].

On the other hand, TAE is a simple procedure with non-chemotherapeutic effect but comparable outcomes to c-TACE. In a recent systematic review and metanalysis, Lawson et al. [12] have found no difference between c-TACE and TAE in terms of OS, progression free survival (PFS) and adverse events, reopening the question whether a chemotherapeutic agent is necessary for a significant tumor response. Nevertheless, comparing c-TACE with TAE may not be the best method to assess differences, as gelatin sponge is not the ideal embolizing agent to induce ischemia. In contrast, DEM-TACE uses permanent particles that induce permanent embolization and chemotherapeutic release within the lesion.

Multiple works have studied the difference between c-TACE versus TAE [13, 14] and DEM-TACE versus c-TACE[11, 15–19], nevertheless, there are only few studies that compared DEM-TACE and TAE with completely different outcomes. Cathomas et al. [20], in their retrospective study, found both treatments as equally effective and safe for very early and early HCC not amenable for resection or ablation, with no disparities in local tumor control and OS. As opposed to the former study, the clinical trial of Malgari et al. [21], demonstrated a clear advantage of DEM-TACE in terms of TTP, with a similar safety profile. However, they acknowledged a significant drawback regarding particle size, as the particles used in their study were quite heterogeneous, ranging from middle to large sizes (300 microns or above), which are not ideal for inducing tissue ischemia. On the contrary, Brown et al. [22] in a more recent clinical trial, did not find any statistically significant differences between the two groups in terms of OS, nevertheless, their study was also questioned due to the inclusion of one-third of patients with BCLC Stage C, a group not considered ideal for TACE or TAE. In contrast to previous studies, we used only 100-micron particles in all patients, enhancing the ischemic component of treatment and reducing heterogenicity, and we also included only patients treatable with TACE/TAE according to m-HAP-II, UNOS/TNM criteria and BCLC guidelines [4].

Futhermore, it is essential to acknowledge the significance of TTP as an endpoint in HCC trials. TTP represents the time interval from the initiation of treatment to the detection of tumor progression or recurrence. In the context of HCC management, prolonging TTP is a critical goal as it correlates with improved patient outcomes, including OS and quality of life (QoL).

Although these results are not in line with the ones found in clinical trial of Malgari et al. [21] this might also be explained by different patient selection, sample size and especially mean tumor dimension which was by far smaller in our study. Despite the theoretical advantages of DEM- TACE, our findings suggest that these benefits may not lead to a significant difference in TTP compared to conventional TAE, especially in small nodules.

In contrast to our findings, a previous study reported a significant difference in hospitalization duration between C-TACE and DEM-TACE with the latter associated with shorter hospital stays [23]. Our study, however, did not demonstrate any statistically significant difference in hospitalization days between the two treatment modalities. This discrepancy raises important questions regarding the purported advantages of DEM-TACE, particularly in terms of cost-effectiveness. Given the higher material costs associated with DEM-TACE, the absence of a reduction in hospital stay in our cohort challenges the assumption that DEM-TACE offers superior economic benefit over other treatments, underscoring the need for further comparative studies to better define its value in routine clinical practice.

Additionally, SAEs are an important consideration in the selection of treatment modalities for HCC, as they can significantly impact patient QoL and treatment compliance. In our study, we did not find any statistical significance between both procedures regarding SAEs comparable to that reported by Malgari et al. and Brown et al.[21, 22] Nevertheless, SAEs differences are very difficult to assess in clinical trials as being quite rare in these procedures, therefore systematic reviews and metanalyses are more suited for this manner. In fact, a meta-analysis of five trials revealed a higher risk of severe adverse events with TACE (cTACE or DEM-TACE) compared to TAE (RR = 1.33, 1.03–1.73, *p* = 0.03), but no significant difference in the 2-year survival rate (RR = 0.88, 0.74–1.06, *p* = 0.18) [24].

Despite the evidence presented in this trial, several limitations warrant consideration. A major limitation is that the study did not reach the planned sample size as specified in the study protocol, which reduces the statistical power and limits the ability to strongly demonstrate equivalence between treatments. Additionally, tumor response and disease progression were assessed locally at each participating center without centralized or blinded image review, which reduces the robustness and reproducibility of the imaging-based primary endpoint (TTP) in a randomized clinical trial setting. Additionally, safety evaluation remains incomplete, as only serious adverse events were reclassified according to CIRSE standards while minor complications were not prospectively collected or analyzed, limiting a comprehensive comparison of safety profiles between treatment arms.. Furthermore, this trial only studied one chemotherapeutic agent, so potential differences between DEM-TACE and TAE using other agents cannot be excluded. Future research endeavors should address these limitations by conducting larger-scale prospective studies with extended follow-up periods, standardized treatment protocols, and different chemotherapeutic agents to provide more definitive conclusions regarding the comparative efficacy of TAE and DEM-TACE.

## Conclusion

In this randomized clinical trial, TAE showed comparable outcomes to doxorubicin DEM-TACE with small particles in the treatment of unresectable early and intermediate stage HCC. These findings suggest that TAE could be considered as an equal alternative for treating HCC with no differences in terms of safety and efficacy.

## Supplementary Information

Below is the link to the electronic supplementary material.

Supplementary file1 (DOCX 16 KB)
