# Supplementary material for: Transarterial Embolization Alone Versus Drug-Eluting Microspheres Chemoembolization for Hepatocellular Carcinoma (RAD-18-TAcE): A Multicenter Randomized Clinical Trial
Source: Cardiovasc Intervent Radiol. 2026 Jun 9;49(10):2052–61. doi: 10.1007/s00270-026-04487-3 (PMC13642458; doi:10.1007/s00270-026-04487-3)
Supplement: Supplementary file 1 [file 270_2026_4487_MOESM1_ESM.docx]

**Supplementary Material – Retrospective Data Collection on Minor Complications**

To complement the prospective analysis, a retrospective review of minor complications was performed in patients included in the trial and treated with Drug-Eluting Microsphere Transarterial Chemoembolization (DEM-TACE) and Transarterial Embolization (TAE). Minor complications were defined according to CIRSE complications classification and included self-limiting clinical events not requiring major therapeutic intervention.

The retrospective dataset was analyzed to explore potential differences in the incidence of mild post-procedural adverse events between the two treatment groups. Statistical comparison did not demonstrate any significant difference between DEM-TACE and TAE in terms of the rate of mild complications (Table 1).

These findings support the safety profile of both procedures and are consistent with the results observed in the prospective cohort, suggesting that the occurrence of minor post-procedural events is not statistically different between the two treatment strategies.

|  | | DEB-TACE | TAE | Total | P-value |
| --- | --- | --- | --- | --- | --- |
| **Vomit** | Yes | 1 | 1 | 2 | 1.000 |
|  | No | 54 | 55 | 109 |  |
|  | Total | 55 | 56 | 111 |  |
| **Pain** | Yes | 4 | 4 | 8 | 1.000 |
|  | No | 51 | 52 | 103 |  |
|  | Total | 55 | 56 | 111 |  |
| **Fever** | Yes | 3 | 4 | 7 | 1.000 |
|  | No | 52 | 52 | 104 |  |
|  | Total | 55 | 56 | 111 |  |

Table 1 adverse events distribution between both groups
